# *Drosophila melanogaster* larvae generate force to counteract external mechanical pressure

**DOI:** 10.1242/jeb.250849

**Published:** 2026-03-10

**Authors:** Yimiao Ding, Yang Lu, Guohua Zhao, Zhefeng Gong

**Affiliations:** ^1^ Department of Neurology of the Fourth Hospital and School of Brain Science and Brain Medicine, Zhejiang University School of Medicine, Hangzhou 310058, China; ^2^ Liangzhu Laboratory, MOE Frontier Science Centre for Brain Science and Brain-Machine Integration, State Key Laboratory of Brain-Machine Intelligence, Zhejiang University, 1369 West Wenyi Road, Hangzhou 311121, China; ^3^ NHC and CAMS Key Laboratory of Medical Neurobiology, Zhejiang University, Hangzhou 310058, China; ^4^ The First Affiliated Hospital, Zhejiang University School of Medicine, Hangzhou 310003, China; ^5^ Department of Neurology, Brain Center, The Fourth Affiliated Hospital of School of Medicine, and International School of Medicine, International Institutes of Medicine, Zhejiang University, Yiwu 322000, China

**Keywords:** *Drosophila* larvae, Mechanical pressure, Counteraction

## Abstract

To counteract or to retreat presents a fundamental dilemma for biological organisms when facing adverse abiotic environmental conditions. In many cases, the predominant strategy animals adopt is to retreat. However, whether counteraction is possible and how the choice between counteraction and retreat is decided are not clear. Here, we report that *Drosophila melanogaster* larvae can actively counter external mechanical pressure, inspired by *Drosophila* larval cleft-squeezing behaviour. We developed a behavioural paradigm to investigate the counteracting force of larvae in response to external pressure. Instead of retreating by crawling backward, some *D. melanogaster* larvae could crawl forward and act against the external physical pressure. Under externally applied forces of 25 mN, 93.9% of forward peristaltic movements increased the counteracting force, while 88.2% of backward peristaltic movements decreased it. The active nature of the counteracting force was reflected by a longer inter-wave delay, more oscillation work and a longer force wave period during consecutive forward peristaltic waves. As the external force was increased from 25 mN to 50, 75 and 100 mN, counteraction by forward peristalsis became less frequent, while retreat by backward peristalsis was more frequent. A reduction of the external pressure immediately following the counteracting forward peristalsis, which might serve as rewarding signal, reinforced the counteraction and induced more forward peristalsis. The rewarding effect of reducing external pressure by forward crawling was much greater than that produced by backward crawling. Our study sheds light on the intricate mechanisms underlying animal proactive responses to adverse abiotic environmental conditions.

## Teaser

**Highlighted Article:** Fruitfly larvae are able to counteract external mechanical forces, demonstrating the animals' innate ability to relieve environmental mechanical pressure.

## INTRODUCTION

Animals adapt to adverse environmental changes largely in three ways: (1) by leaving the adverse environment and relocating to a suitable new environment, as in seasonal migration; (2) by staying in the same environment but changing their way of life, as in hibernation or adaptation of organs or metabolism; or (3) by counteracting and changing local environmental conditions and making them suitable for living (Lee, 1985; Evans et al., 2016; Roberts et al., 2019; Buarque de Macedo et al., 2021). Usually, pressure from the environment is huge and largely unchangeable, so it is more common to observe the adaptation of animals to environmental changes in the first and second way. However, if the environmental pressure is mild and the animals are powerful enough, it is possible for it to change the local environment by its active or ‘intentional’ behaviour.

One well-known example of an animal's active counteraction of environmental pressures is the beaver. During flood or drought, beavers do not leave for a new living place or change their behaviour. Rather, they build dams to slow down the flow and create a wetland habitat suitable for their lodge. Beavers are thus called ‘ecosystem engineers’ (Wright and Jones, 2006; Fedyń et al., 2024). Similar to beavers, earthworms are also ecosystem engineers (Wright and Jones, 2006; Rahman and Candolin, 2022). Earthworm can dig into hard earth and make the earth loose enough for them to live in. Some animals demonstrate active counteracting behaviour against a seemingly adverse environmental pressure even when they are unable to change it. For example, fish such as salmon and trout swim upstream against the current, which is not affected by the upstream swimming of the fish. Although there have been reports that fish swim upstream for more fresh air or because of natal philopatry, it is apparent that these fish have the innate ability to counteract the physical pressure of the water flow (Cava et al., 2016).

All these examples show that animals can possess the innate ability to counteract environmental pressure in order to change the local environment to a condition suitable for them to live in. This is echoed by observations from *Drosophila* larvae, which like to squeeze into the cleft between food and the wall of the food vial (Kim et al., 2017). During this procedure, the larva needs to overcome the mechanical pressure from the cleft and tug its body in. There have been few studies on counteraction against environmental pressure by animals in a laboratory setting (Sun et al., 2022); thus, the cleft-burrowing behaviour of the class model animal *Drosophila* larvae provides an opportunity to study this phenomenon. In line with this, we developed a behavioural paradigm to investigate whether *Drosophila* larvae possess the ability to act against physical environmental pressure, how the active counteracting force against external physical pressure is exerted, and how the behaviour is modulated by the change in external pressure. This concept relates to the broader phenomenon of active control, where organisms exhibit adaptive responses to environmental pressure, potentially involving the modulation of their behaviour based on experience. While animals often react to external forces through instinctual behaviours, repeated exposure may enable them to refine their strategies over time, suggesting a form of learning that influences their ability to counteract. In the case of *Drosophila* larvae, their ability to modulate their counteraction efforts under varying pressures may reflect such adaptive processes. By controlling the external physical pressure, which is hard to do in a wild environment, we found that *D. melanogaster* larvae did actively act against external mechanical pressure, with the counteraction endeavour negatively related to external pressure. Notably, artificially reducing external pressure following counteracting or retreating behaviour served as a reward to encourage behavioural repetition, while increasing the external pressure serve as a deterrent.

## MATERIALS AND METHODS

### Fly strains and husbandry

All flies were raised at 25°C on standard medium and a 12 h:12 h light:dark cycle (Zhao et al., 2024). The Canton-S wild-type strain was used in this work.

### Experimental setup for measuring *D. melanogaster* counteractive behaviour against physical pressure

To simulate the cleft between the vial wall and food encountered by *D. melanogaster* larvae during digging behaviour, a custom-made apparatus integrating biomechanical force measurement and real-time imaging of larval behaviour was developed (Movie 1). The system comprised the following parts: (1) a high-precision plate-type force sensor (RDF-DP01, RLD Inc.) positioned perpendicular to the larval dorsoventral axis for real-time vertical force quantification; (2) a *z*-axis micro-displacement platform (travel resolution: 10 μm), controlled via a MATLAB R2024a-based proprietary program, with environmental pressure dynamically adjusted by modulating the height of an overhead coverslip; (3) control interface-enabled serial communication with both the force sensor and the displacement platform, allowing synchronized initiation of baseline force (5 s ramp phase) through programmable coverslip descent; and (4) an industrial camera (Minsvision-SUA630C) mounted above to record larval locomotion. The geometry of the cleft was mechanically tuned using differential-height fastening screws to create adjustable apex angles, replicating gradient confinement conditions. We used a near-3 deg tilt as our standard configuration. The counteracting force and locomotion behaviour of the larva were respectively recorded by the force sensor and the camera at a sampling rate of 5000 Hz and 136 frames s^−1^ at a resolution of 1280×1024 pixels simultaneously. Force recoding and force feedback control were done by our custom MATLAB App Forcecal (https://github.com/DingYimiao/Gonglab).

### Measurement of larval active counteracting force

Mid-to-later third-instar larvae were selectively harvested using soft-bristle brushes, rinsed in deionized water to remove food, and briefly blotted on lint-free lens paper to standardize cuticular adhesion. A single larva was positioned upright on a rigid aluminium alloy platform integrated with the force sensor, with the head oriented toward the narrow side of the cleft and with the tail toward the opening side of the cleft. The rigid aluminium alloy platform was used to prevent confounding effects from substrate deformation. Following a 30 s acclimation period for the larvae to recover from handling stress and initiate spontaneous crawling, the coverslip was lowered to apply initial baseline-level confinement force (sensor validated) coinciding with larval peristaltic initiation. After the measured force reached the set baseline level (25/50/75/100 mN in the following experiments), the platform was locked in place without further controlled displacement and all subsequent force change originated purely from the larva's movements against the fixed constraint. The micro-displacement platform operates in closed-loop control at a refresh rate of 5 Hz, achieving <10 μm positioning accuracy within 200 ms response time, by our custom MATLAB App Forcecal (https://github.com/DingYimiao/Gonglab).

The 120 s observation window began immediately after achieving stable cephalocaudal alignment (head–tail angle <10 deg). Continuous force telemetry and dual-view video recordings were obtained throughout each trial. The trial was terminated if the force was less than 5 mN (indicating a loss of mechanical engagement) or if the head reorientation angle relative to the slit axis exceeded 45 deg. All trials were performed under 65% relative humidity and a temperature of 25±0.5°C. Under each experimental condition of 25/50/75/100 mN baseline level force, 40 larvae were tested. Each larva was used only once.

### Training *D. melanogaster* larvae to induce active counteraction

The training programme was realized with our custom MATLAB-programmed *z*-axis precision displacement stage integrated with a force sensor for real-time force recording and feedback control. During training, each larva was subjected to a defined initial force of 100 mN. To induce enhanced forward peristalsis, each forward peristaltic wave triggered a 20 mN decrease in force, while each backward wave triggered a 20 mN increase in force, immediately following the application of the initial 100 mN force. This feedback loop continued until the measured force was lowered to 0 mN. For each larva, the entire session was repeated twice. To induce enhanced backward peristalsis, each forward wave triggered a 20 mN increase in force, while each backward wave triggered a 20 mN decrease in force, immediately following the initial 100 mN force. This feedback loop also continued until the environmental force dropped to 0 mN. The entire session was repeated twice for each larva. Larvae were excluded from training for enhanced forward peristalsis if they exhibited ≥5 consecutive backward waves without forward movement after pressure increase, or if they showed no peristaltic activity under pressure. Larvae were excluded from backward escape training if they showed no peristaltic activity waves under pressure. Larvae not subjected to training were allowed to crawl freely on the platform for 2 min and then directly used in the test session as described below.

Immediately after the two training sessions, the larvae were subjected to a test session to evaluate the impact of training on larval locomotion direction. In a test session, each larva was tested only once under a 25 mN initial force and allowed to crawl forward or backward for 120 s, using the same protocol as described in ‘Measurement of larval active counteracting force’, above. The number of forward and backward peristaltic waves was counted. Ten larvae were used for each group subjected to the training sessions followed by one test session.

### Quantification of larval locomotion and counteracting force

The start and end time of a larval forward peristaltic wave were judged by the time that segment A8 (tail) began to contract and the time when segment T1 (head) relaxed. The start and end time of a backward peristaltic wave were judged by the time that the larval head began to contract and the tail segment relaxed. The waves in the curves of force generated during peristalsis were considered to be corresponding force waves.

Peaks and troughs of force waves were identified using the *findpeaks* function in MATLAB. For peak detection, a sliding window of 5 s was specified. The parameter of minimum peak prominence was set to 5 mN to filter out insufficiently prominent peaks and the other parameter minimum peak distance was set to 3 s to merge or ignore overly close peaks while retaining the highest one. The same parameters were applied for wave trough detection. The start time of a wave period was defined as either the first local minimum following the preceding peak or the last local minimum preceding the current peak. The end time of a wave period was determined when both the curve slope magnitude remained <0.5 mN s^−1^ for ≥0.5 s and the overlap between two adjacent wave periods was <10%, to expose and identify the trough in between.

For quantitative analysis of force waves and consecutive force waves, the force waves were judged to be consecutive only if the direction of peristalsis was the same and the interval between neighbouring waves was less than 30 s. The following parameters were used to describe the force waves: (1) force change between two consecutive peristaltic waves – the difference between the peak value of two adjacent force waves; (2) force change per bout of consecutive waves – the difference between the peak values of the current wave and the initial wave; (3) amplitude of a force wave – the difference between the peak value and the end value of the wave; (4) duration of a force wave – the time span between the start and end of a single force wave; (5) inter-wave delay – the time interval between the end of the previous wave and the start of the following wave; and (6) work of a force wave – estimated by the formula (*f*_max_^2^*−f*_min_^2^)/2*k*, where *f*_max_ and *f*_min_ are respectively the maximum and minimum value of the force wave and *k* is the elastic coefficient of the larval body. *k* was obtained from the force–displacement correlation (Fig. S1).

To illustrate how the counteracting force waves change with the increase in the number of consecutive force waves, we performed normalization for each parameter as follows:
(1)

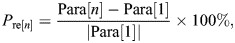
where *P*_re[*n*]_ is the rescaled value measuring the change in the parameter relative to the absolute value of the first wave, and Para[*n*] and Para[1] are the value of the *n*th wave and the first wave, respectively.

### Statistics

Statistics were calculated with Prism 9.5 (https://www.graphpad.com/). For all experiments, unpaired *t*-test was used to assess the difference between forward and backward peristalsis. Kruskal–Wallis test with Dunn's *post hoc* test was used to assess the difference among different external pressures. One-way or two-way ANOVA with Šidák’s multiple comparisons test was used for non-parametric statistics.

For analysis of the correlation between locomotion mode and counteracting force, behavioural kinematics and vertical force dynamics were temporally aligned using frame-accurate time stamps (0.5 s synchronization error tolerance) prior to correlation analysis. Raw locomotion traces were classified into three discrete states: forward peristalsis (coded as 1), static (0) and backward peristalsis (−1). Force signals from the normalized matrix underwent baseline subtraction using the initial 5 s pre-compression phase as reference. To quantify state-specific mechanical coupling, we implemented a point-biserial correlation framework in MATLAB R2024a. For each specimen (*n*=40), paired behaviour–locomotion vectors were truncated at the last valid indices of both datasets, ensuring temporal congruence. The correlation coefficient *r*_pb_ for each locomotor state (forward/backward) was computed as:
(2)

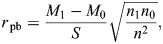
where *M*_1_ and *M*_0_ represent mean force magnitude during active states (forward: 1, backward: −1), respectively; *S* denotes the pooled standard deviation of force measurements; and *n*_1_, *n*_0_ and *n* correspond to frame counts of active/static states and total valid frames.

### Code availability

The custom-written MATLAB App Forcecal code, performing real-time force monitoring, precision force modulation, synchronized behaviour recording and data processing, is available from GitHub (https://github.com/DingYimiao/Gonglab). The documents (Forcecal: *Drosophila* Larval Force Measurement System) detailing the architecture of the MATLAB App, hardware integration and validation metrics are also included.

## RESULTS

### A behavioural paradigm for studying the action of *D. melanogaster* larvae against external mechanical pressure

**Fig. 1.**
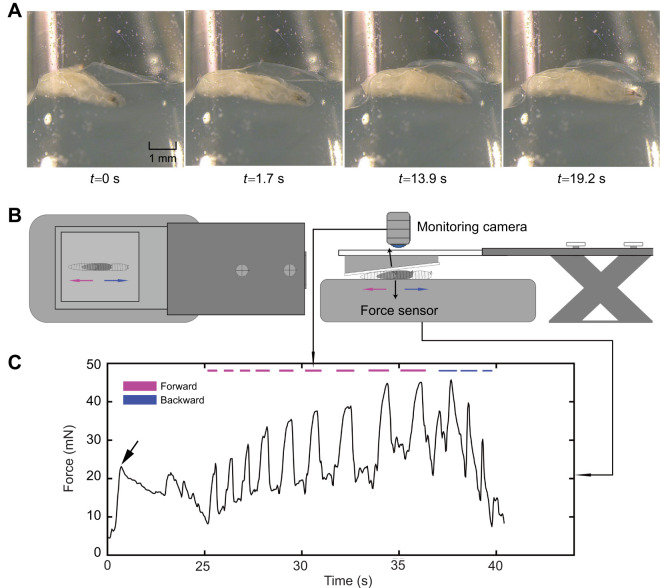
***Drosophila melanogaster* larvae have the ability to actively overcome mechanical pressure.** (A) Time lapse of a larva digging into 3% agarose by forward peristalsis. (B) Diagram of the setup for studying the counteractive behaviour of a larva that squeezed into an artificial cleft. The force sensor below the larva simultaneously records the force exerted by the larvae. The locomotion behaviour of the larva is simultaneously recorded by the monitoring camera. (C) A sample curve of the counteracting force signal of a larva loaded initially with 25 mN normal force. The force trace begins after the acclimation period, with an initial ramp up to a baseline level (25 mN in this sample, indicated by the arrow). Subsequent fluctuations reflect larval active responses against this fixed constraint. The time period of forward crawling is shown in purple and that of backward crawling is shown in blue.

According to observations from daily fly husbandry, wandering *D. melanogaster* third instar larvae like to squeeze into the cleft between food and the wall of food vial (Kim et al., 2017). The same behaviour could be observed on an agar plate. Although larvae sometimes chewed the agarose gel to make space, they also often tried to expand the existing cleft to allow them to squeeze in (Fig. 1A). The most common action of larval cleft-squeezing behaviour was the sequential axial contraction of the body to expand the gap.

To directly measure the counteracting force of the larva against the external physical pressure, and to better control the external force exerted on the larva, we developed a setup to simulate *Drosophila* larval cleft squeezing behaviour (Fig. 1B). During the test, a larva was placed on the platform of a force sensor and then covered with a coverslip at an angle to form a cleft between the platform and coverslip. The height of the coverslip was adjusted to apply different initial pressures on the larva. The larva was oriented with its head towards the narrower side of the cleft. The larva could crawl forward towards the narrower side and encounter greater mechanical pressure, or crawl backwards towards the open side and experience reduced pressure. The counteracting expanding force in the vertical direction that the larva exerted on the platform was measured by a force sensor. When the larva crawled, the forces and body movements were simultaneously monitored, allowing direct comparison between body movement and the force generated (Fig. 1C).

### *Drosophila melanogaster* larvae counteract external pressure by forward peristalsis and retreat by backward peristalsis

**Fig. 2.**
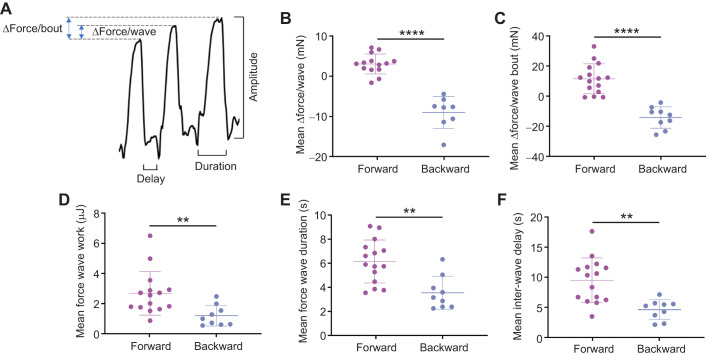
**Forward and backward peristalsis respectively represent active counteraction and retreat behaviour upon application of external physical pressure.** (A) Schematic representation of the parameters describing larval counteracting force. (B–F) The force change between two consecutive peristaltic waves (B), the force change in a bout of consecutive peristaltic waves (C), the amplitude (work) (D) and duration (E) of a force wave, and the inter-wave delay between two neighbouring waves (F), for forward and backward crawling under an initial external force of 25 mN. Each data point represents the mean peristaltic wave value for an individual larva (*n*=15). In some cases, there are multiple data points for the same larva. Bars indicate means±s.d. Asterisks indicate significance (**P<0.01, ****P<0.0001; unpaired *t*-test).

In our experimental setup, larvae exhibited diverse behavioural responses under an initial baseline force of 25 mN. As shown in Fig. 1C, a larva could crawl either forward or backward to generate an oscillating force ranging from less than 10 mN to more than 40 mN. The force curve was described with the five parameters indicated in Fig. 2A.

As illustrated in Fig. 2B, in a 2 min test period with an initial force of 25 mN, 93.9% of forward peristaltic movements increased the counteracting force, while 88.2% of backward peristaltic movements decreased it. On average, the counteracting force increased by about 3 mN after each forward wave and decreased by about 7 mN after each backward wave (Fig. 2B). Continuous forward peristalsis epochs could increase the resisting force by more than 10 mN, while continuous backward peristalsis epochs could decrease the resisting force by more than 10 mN (Fig. 2C). This was expected, as forward peristalsis moves the larva into a narrower space to generate a higher counteracting force while backward peristalsis does the opposite, which was demonstrated by the apparent negative correlation between the measured force and the distance of larvae to the slit (Fig. S1). As the force measured by the sensor reflects both the counteracting force exerted by the larva and the concurrent cleft pressure acting on it, the gradual increase in force suggests that the larvae exerted greater effort to counteract the increased external pressure. Forward peristalsis is probably an attempt to create more space and alleviate the pressure.

A closer observation of larval locomotion and force showed that the average work and duration of forward peristalsis epochs was significantly longer than those for backward peristalsis epochs (Fig. 2D,E). Additionally, the delay between two consecutive forward peristalsis waves was generally larger than that for backward peristalsis (Fig. 2F). These results indicate that forward peristalsis is generally slower, and mechanically more challenging than backward peristalsis under external physical pressure.

Taken together, when facing external physical pressure, *D. melanogaster* larvae adopt forward peristalsis as the behavioural strategy to generate a larger counteracting force against external pressure, and backward peristalsis as the strategy to retreat from the pressure.

### Consecutive peristaltic waves generate cumulative effects on counteracting forces

**Fig. 3.**
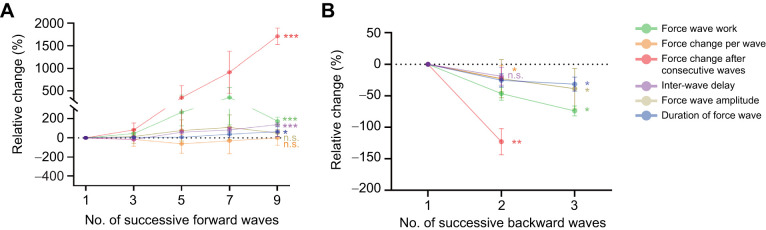
**Consecutive forward or backward peristaltic waves have a cumulative effect on counteracting forces.** The five parameters of force change between two consecutive peristaltic waves – force change after consecutive waves, force wave amplitude, force wave work, inter-wave delay and duration of force wave – change as the number of consecutive forward (A) and backward (B) peristaltic waves increases. Inter-wave changes are the difference between the parameter value of that wave and that of the next wave. Because larvae move out of the arena after three consecutive backward waves, there are only two waves for force change per wave, force change after consecutive waves and inter-wave delay in B. Bars indicate means±s.d.; *n*=15 for all groups. Asterisks indicate significance (*P<0.05, **P<0.01, ***P<0.001; n.s., not significant; one-way ANOVA).

Although forward and backward peristalsis were respectively correlated with an increase and decrease in counteracting force, it was still not clear whether such a resisting force is active or passive. In fact, a single wave of forward peristalsis did not always result in an increase in resisting force (see typical example in Fig. 1C), although 93.94% of forward waves increased force, with 88.24% of backward waves decreasing resistance (Fig. 2B). We proposed that if a larva persistently moved forward against increasing external pressure, it was more likely that its action against the external pressure was active. As shown in Fig. 3A, consecutive forward peristaltic waves progressively amplified counteracting force through sustained force buildup, indicating that the larvae were trying to push against the glass coverslip. In contrast, backward peristaltic waves reduced both mechanical resistance and action duration, showing that the larvae were trying to alleviate the pressure and escape (Fig. 3B).

The active nature of the counteracting force was also reflected by the force curves after multiple consecutive waves. As the wave number increased, the average delay between two consecutive forward peristalsis waves increased significantly (Fig. 3A), while that of backward peristaltic wave decreased although only relatively subtly (Fig. 3B). Similar trends were observed for the oscillation work and duration of force waves, for both consecutive forward and backward peristalsis (Fig. 3). Obviously, the increase in the counteracting force and the simultaneous increase in external pressure during forward peristalsis made larval forward crawling more difficult, indicating that forward crawling, or at least consecutive forward crawling, was an active larval endeavour to counteract the external pressure. What makes this ‘active’ in our interpretation is that the larvae persisted in forward pushing even as the resistance increased and, crucially, they learnt to amplify this effort when it led to pressure relief (as shown in our training experiments). This is not just mechanical work – it is the behavioural signature of an organism strategically investing more effort when it counts, which is why we think it reflects true active counteraction rather than simple movement.

### *Drosophila melanogaster* larvae active counteraction is more significant against milder external pressure

We next tried to find out the effect of external pressure strength on larval counteractive behaviour. We tried initial forces ranging from 25 mN to 50, 75 and 100 mN, under which larvae were not pressed too tightly so that they could not move, or too loosely such that they could easily escape the testing arena.

**Fig. 4.**
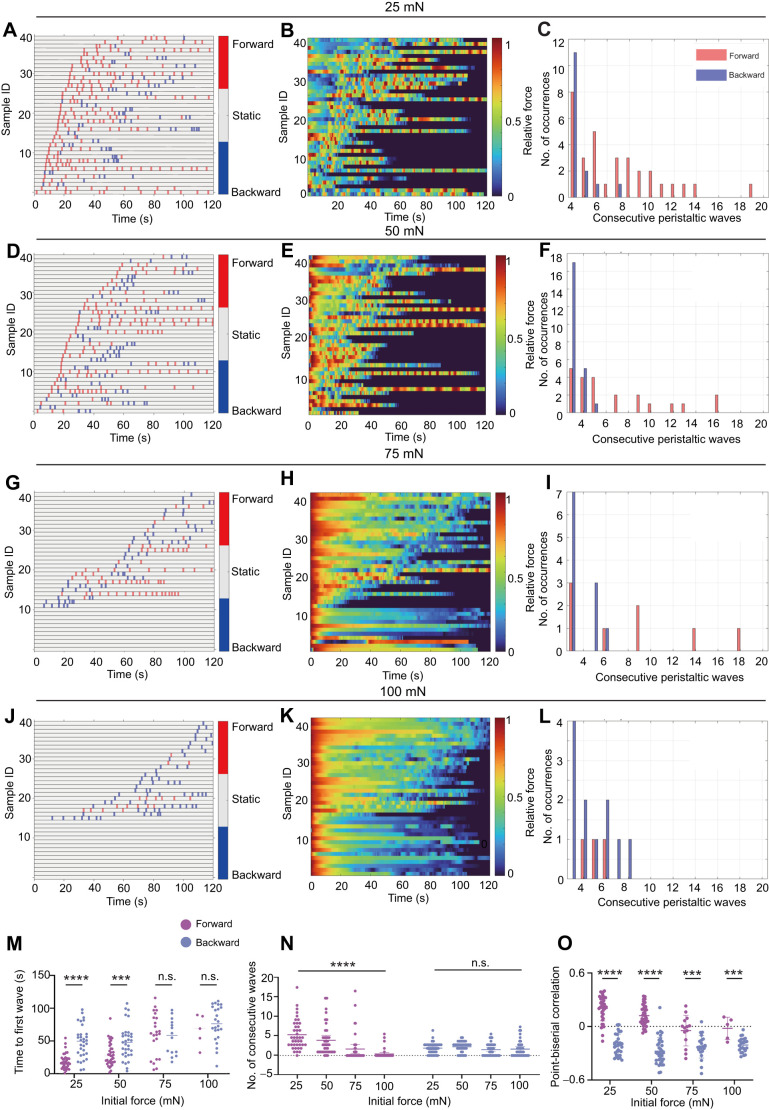
**Larval counteractive behaviour is more significant against milder external pressure.** (A–L) Raster plot of forward and backward peristalsis, heatmap of the corresponding *z*-normalized counteracting force and frequency of consecutive peristaltic waves, under a force of 25 mN (A−C), 50 mN (D–F), 75 mN(G–I) and 100 mN (J–L). (M) Time to occurrence of the first peristaltic forward and backward waves under initial forces of 25, 50, 75 and 100 mN. The higher the initial force, the later and lower the number of forward peristaltic waves. (N) The number of consecutive forward peristaltic waves is reduced under higher force but that of consecutive backward peristaltic wave is not affected. (O) Point-biserial correlation analysis between locomotion mode and continuous force signals. The dotted reference line denotes chance-level correlation. As the initial force increases, the correlation between force and forward peristalsis decreases, while the correlation between force and backward peristalsis is not changed. Each point represents one larva. Error bars represent s.d. in N and s.e.m. in M and O; *n*=40 for each group in all panels. Asterisks indicate significance (***P<0.001, ****P<0.0001; n.s., not significant; Kruskal–Wallis test with Dunn's *post hoc* test).

Most larvae initiated locomotion by forward peristalsis under forces of 25 and 50 mN (Fig. 4A,D,M). But at higher forces of 75 and 100 mN, more larvae started with backward peristalsis (Fig. 4G,J,M). This probably means that larvae prefer to act against lower pressures than against higher pressures. As the pressure increased, the delay for the first peristaltic wave to occur increased while the number of peristaltic waves decreased for both forward and backward peristalsis (Fig. 4A,D,G,J). About 23% of all tested larvae showed complete behavioural arrest under 75 and 100 mN. This could be due to either the increased friction force that mechanically limited larval movement under higher pressures or the physiological inhibition of the larval neural circuit that commands movement by the higher pressure.

We noted that under an initial force of 25 mN, larvae showed a higher probability of forward crawling for the whole duration of the test (Fig. 4A,C), but under a high initial force of 100 mN, the larvae showed more backward crawling despite the force decreasing later (Fig. 4J,L). This means that low pressures facilitated forward crawling while higher pressures triggered more backwards escaping attempts. In line with the locomotion patterns, larval counteracting force was more likely to maintain a non-declining trend under low initial forces, while it showed a consistent declining trend under high initial forces (Fig. 4B,E,H,K). As initial force increased, the frequency of long-bout consecutive forward peristalsis decreased (Fig. 4C,F,I,L,N). This was probably because pressure was increased by consecutive forward crawling and, in return, prevented forward crawling. In contrast, long-bout backward peristalsis was obviously less frequent, especially for bouts of more than 10 consecutive waves. This is because larvae could be pushed out of the test arena by a few backward peristaltic waves so that number of consecutive backward waves was limited. Furthermore, the positive correlation between forward peristalsis and the counteracting force declined as initial external pressure increased (Fig. 4O). This was probably because under higher pressure, the deformation of the larval body during forward crawling was restricted so that the generation of counteracting force was prevented. Interestingly, the negative correlation between backward peristalsis and counteracting force was unaffected by higher pressures, suggesting that backward crawling was insensitive to high pressure with respective to reducing counteracting force.

Taking these results together and largely based on the relationship between increased counteracting force and forward crawling, we conclude that *D. melanogaster* larval active counteraction was more significant against milder external pressures and was weaker against higher external pressures.

### Larval counteractive behaviour is enhanced by rewarding larvae with pressure alleviation

**Fig. 5.**
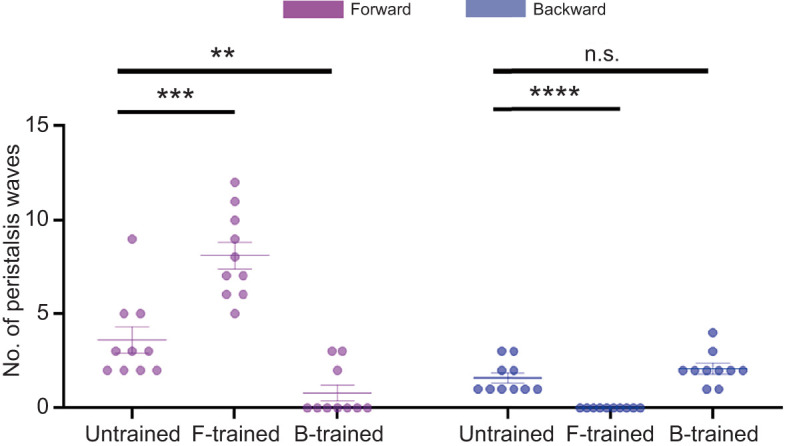
**Larval counteractive behaviour is enhanced by force reduction and inhibited by force increase.** In forward-trained (F-trained) larvae, forward peristalsis was followed by a force decrease and backward peristalsis was followed by force increase; in backward-trained (B-trained) larvae, backward peristalsis was followed by force decrease and forward peristalsis was followed by a force increase. Asterisks indicate significance (**P<0.01, ***P<0.001, ****P<0.0001; n.s., not significant; two-way ANOVA with Šidák's multiple comparisons test). Bars indicate means±s.d.; *n*=10 for each group.

As the tests usually ended up with larval backward peristalsis to escape from the external pressure (Fig. 4A), we wondered whether backward crawling and thus the decrease in force were rewarding while forward crawling and the increase in force served as punishment. We therefore tested whether an artificially imposed reward or punishment could encourage larvae to counteract the external force or to retreat. We designed training programmes for the larvae such that external force was increased or decreased immediately following a forward or backward peristaltic wave (see Materials and Methods). Larvae receiving a reward after forward peristalsis showed more forward crawling than naive controls, and those rewarded for backward movement showed less forward crawling than naive control groups (Fig. 5). In contrast, backward movement-rewarded groups did not show a change in backward peristalsis compared with naive groups, while larvae receiving a reward after forward peristalsis showed less backward crawling (Fig. 5). These results suggested that a reduction of force was indeed rewarding and an increase of force was indeed punishing, in support of our previous conclusion that continuous forward peristaltic waves indicated larval active counteraction against punishment. Furthermore, the increase in the number of forward peristaltic waves in pro-forward training was obviously much more than that of backward peristaltic waves in pro-backward training (Fig. 5), indicating that the rewarding effect resulting from overcoming pressure was much more than the rewarding effect obtained from retreating. The preferential increase in forward crawling after rewarded trials (Fig. 5) indicates learned association rather than mechanical liberation, as larvae remained mobile even at 100 mN force. This reward-specific behavioural modification suggests strategic counteraction rather than simple motion restoration.

## DISCUSSION

In this work, we established an experimental paradigm to test *D. melanogaster* larva's ability to actively counteract external mechanical force. We simultaneously measured the larval counteracting force against pressure applied from the upper front, and monitored the effect on larval locomotion behaviour. Larvae demonstrated either counteractive behaviour by forward crawling to increase the force or retreat by backward crawling to reduce the force. It was found that larvae were more likely to show counteractive behaviour when the external pressure was milder. Interestingly, active counteraction was more likely to re-occur if reduced pressure ensued and is less likely to occur if increased external pressure ensued. Our results suggest that even invertebrates such as *D. melanogaster* larvae have the innate ability to actively counteract external force from the environment.

We found that the increasing counteracting force against external pressure was mainly generated by forward peristalsis, while backward peristalsis only led to a reduced counteracting force. Why is the counteracting force so different in backward and forward crawling? This is seemingly because the larva was placed towards the narrower side of the cleft created by the coverslip, so that forward crawling pushed it deeper into the cleft while backward crawling moved the larva to the more spacious side of the cleft as the coverslip was at a slight angle (∼3 deg). Even if the coverslip was held horizontal, the measured counteracting force still increased during forward crawling and decreased during backward crawling. However, compared with the small directional force bias (forward: 18.94±5.51 mN versus backward: 16.21±3.68 mN) under a horizontal coverslip, the 3 deg tilt amplified this difference (37.59±17.02 mN forward versus 18.78±8.89 mN backward) and better mimicked natural cleft environments (Fig. S2). We assumed that the larval body lifts up during the peristaltic period and the bump formed is not relieved during the inter-period phase (Heckscher et al., 2012; Liu et al., 2023), so that the counteracting force is increasingly larger during continuous forward crawling. Conversely, the larval body is probably more flattened during backward crawling so that the counteracting force is decreased. The fluidic hydrostatic larval body structure counteracts the body deformations during both forward and backward crawling. To resist the body deformation, active muscle contraction may be required during forward crawling to squeeze open the cleft, whereas during backward crawling, the re-thickening of the body is supported by passive recoil of the relaxed muscles and body wall. But how the action of the larval muscle system is differentially organized during forward and backward peristalsis (Zarin et al., 2019) with respect to generating a counteracting force needs further investigation.

We considered the consecutive forward peristalsis against the coverslip indicates the active counteraction of external pressure by the larvae. First, as the position of the platform and the coverslip that sandwiched the larva did not change, the change in measured force came solely from larval movements. The gradual rise in pressure meant that the larva actively chose to bear more pressure as it moved deeper into the constrained space. Second, the peaks of individual force waves, which could increase by 3–5 mN per consecutive wave, also reflected the larva's active counteraction of external pressure. This is comparable to a climber – the increase in elevation comes from the terrain, but the extra bursts of effort (wave peaks) comes from the climber's active pushing. As the timing of the rhythmic force spikes precisely matches that of the larval crawling patterns, and the amplitude of the peaks show progressive amplification during consecutive waves, the rhythmic force spikes are strongly indicative of ‘deliberate’ or active larval counteractive behaviour. Third, as forward peristalsis was promoted by rewarding the larva through the alleviation of pressure, the forward crawling that led to higher external pressure is thus very likely to be active, i.e. the larva was trying to get the reward it was expecting.

One other intriguing question is why some larvae choose forward while others choose backward crawling when subject to external mechanical force? Both strategies, crawling forward to overcome pressure or crawling backward to retreat from it, are practicable. However, while retreat always alleviates external pressure, counteraction may alleviate external pressure completely when it is mild but is more likely to fail when it is strong, which could respectively mean a high-value reward or regular punishment. Between the two choices when facing environmental pressure, the chance to reduce the pressure by counteraction is very low, but the reward can be very high if it is realized – known as contrast effect (Crespi, 1942; Breiter et al., 2001). In contrast, reducing the pressure by retreating is easy but the reward is relatively low. As the rewarding effect resulting from counteractive behaviour is more than the rewarding effect obtained from retreat, it is possible for the larva to show counteractive behaviour in the expectation of a higher value reward (Schultz et al., 1997). If the rewarding effect of counteraction is more than the punishing effect resulting from the increased pressure, the chances of counteraction can be even higher. The animal needs to find a balance between the possible outcomes and make a choice depending on its internal and external conditions.

One other question concerns the similarities and differences between responses against the abiotic environment and those against other animals, such as fighting or aggressive behaviour. The similarity between these two types of counteraction is obvious: animals try to reduce external pressure through active behaviour. The difference is also significant: the pressure from other animals such as predators or competitors is usually more variable and not as persistent and strong as environmental pressures. This could explain, to a certain extent, why animal aggressive and fighting behaviours are often seen while counteractions against abiotic environmental pressure are less reported. In line with this, if we reduce the environmental pressure to a level such that the animals have a chance to overcome or to resist it, they are more likely to show counteractive behaviour. It is conceivable that the neural and molecular basis of the counteractive behaviour against abiotic environmental pressure shares some similarity with that of fighting or aggressive behaviour, but there also must exist some differences between them.

## Supplementary Material

10.1242/jexbio.250849_sup1 Supplementary information
