# Supplementary material for: Drosophila melanogaster larvae generate force to counteract external mechanical pressure
Source: J Exp Biol. 2026 Mar 10;229(5):jeb250849. doi: 10.1242/jeb.250849 (PMC13006524; doi:10.1242/jeb.250849)
Supplement: Supplementary information [file jexbio-229-250849-s1.pdf]

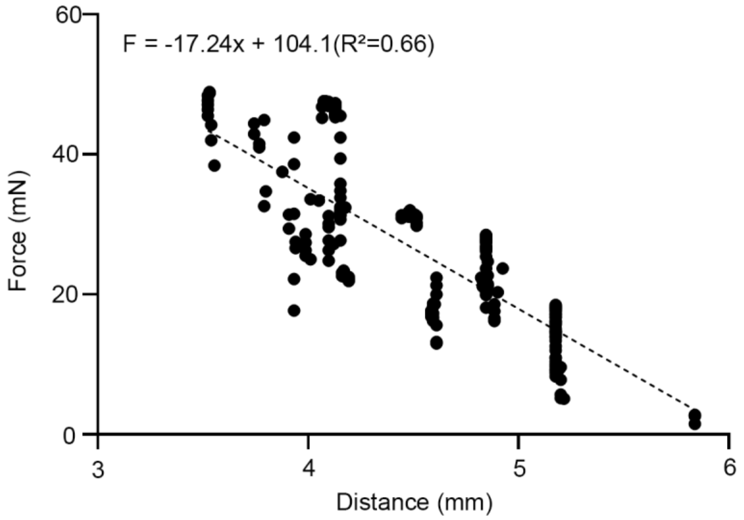

Fig. S1. The relationship between force and larval displacement. Linear regression, F = -17.24x + 104.1, R^2 = 0.66, P<0.0001. n = 224.

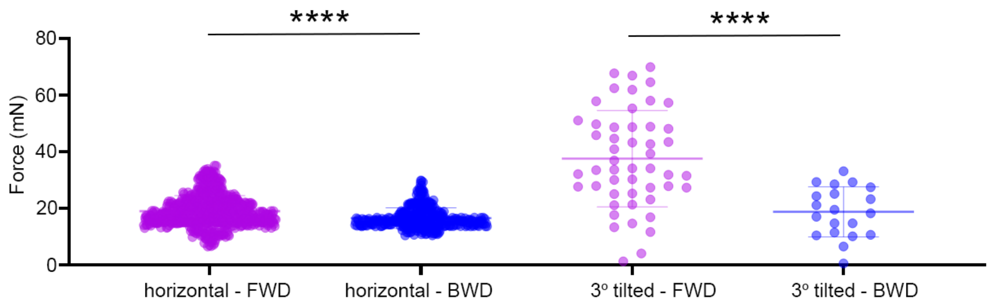

Fig. S2. The counteraction force under force of horizontal and 3° tilted coverslip. n=613, 354, 50 and 20 for horizontal -FWD, horizontal -BWD, 3° tilted FWD, 3° tilted BWD respectively. ****, *p*<0.0001, Mann Whitney test. Bars, Mean±SD.

Journal of Experimental Biology • Supplementary information

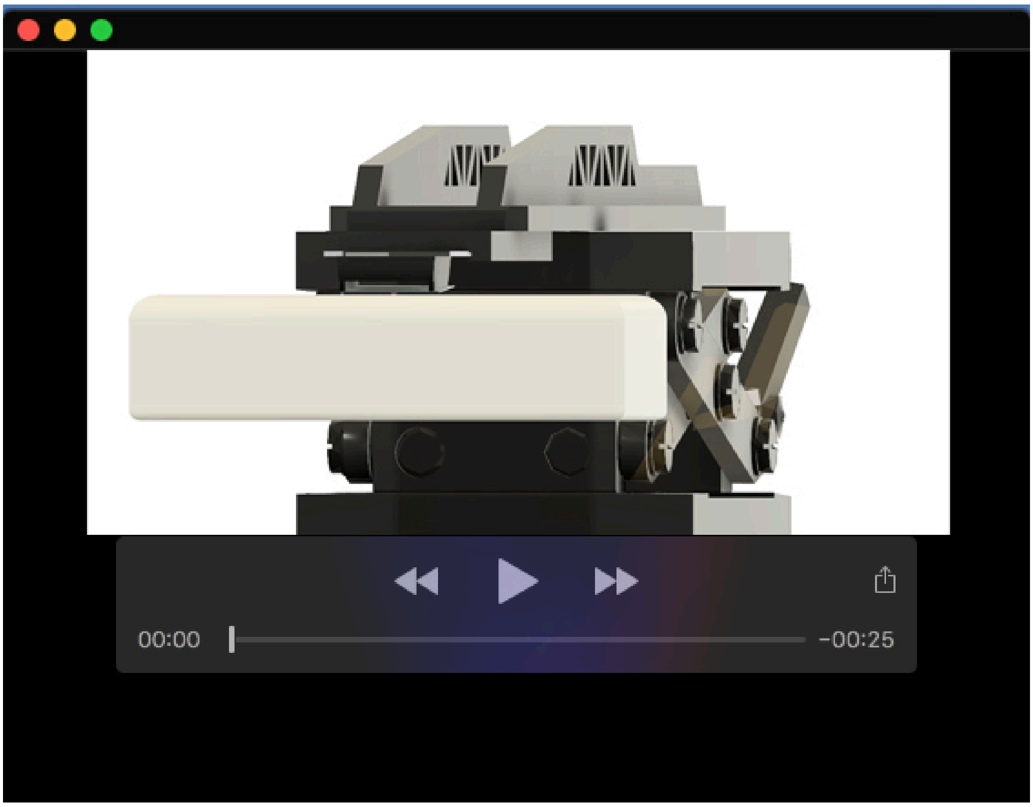

Movie 1. Cartoon of the set up for measuring Drosophila counteraction force against physical force.

Journal of Experimental Biology • Supplementary information
